# Better efficacy in differentiating WHO grade II from III oligodendrogliomas with machine-learning than radiologist’s reading from conventional T1 contrast-enhanced and fluid attenuated inversion recovery images

**DOI:** 10.1186/s12883-020-1613-y

**Published:** 2020-02-07

**Authors:** Sha-Sha Zhao, Xiu-Long Feng, Yu-Chuan Hu, Yu Han, Qiang Tian, Ying-Zhi Sun, Jie Zhang, Xiang-Wei Ge, Si-Chao Cheng, Xiu-Li Li, Li Mao, Shu-Ning Shen, Lin-Feng Yan, Guang-Bin Cui, Wen Wang

**Affiliations:** 1 Department of Radiology & Functional and Molecular Imaging Key Lab of Shaanxi Province, Tangdu Hospital, Air Force Medical University, 569 Xinsi Road, Xi’an, 710038 Shaanxi People’s Republic of China; 2 Student Brigade, Air Force Medical University, Xi’an, 710032 Shaanxi China; 3 Deepwise AI Lab, Deepwise Inc, No.8 Haidian avenue, Sinosteel International Plaza, Beijing, 100080 China; 4 Department of Stomatology, PLA 984 Hospital, Beijing, China

**Keywords:** Oligodendrogliomas, Machine learning, Radiomics, Random forest (RF), Magnetic resonance imaging (MRI)

## Abstract

**Background:**

The medical imaging to differentiate World Health Organization (WHO) grade II (ODG2) from III (ODG3) oligodendrogliomas still remains a challenge. We investigated whether combination of machine leaning with radiomics from conventional T1 contrast-enhanced (T1 CE) and fluid attenuated inversion recovery (FLAIR) magnetic resonance imaging (MRI) offered superior efficacy.

**Methods:**

Thirty-six patients with histologically confirmed ODGs underwent T1 CE and 33 of them underwent FLAIR MR examination before any intervention from January 2015 to July 2017 were retrospectively recruited in the current study. The volume of interest (VOI) covering the whole tumor enhancement were manually drawn on the T1 CE and FLAIR slice by slice using ITK-SNAP and a total of 1072 features were extracted from the VOI using 3-D slicer software. Random forest (RF) algorithm was applied to differentiate ODG2 from ODG3 and the efficacy was tested with 5-fold cross validation. The diagnostic efficacy of radiomics-based machine learning and radiologist’s assessment were also compared.

**Results:**

Nineteen ODG2 and 17 ODG3 were included in this study and ODG3 tended to present with prominent necrosis and nodular/ring-like enhancement (*P* < 0.05). The AUC, ACC, sensitivity, and specificity of radiomics were 0.798, 0.735, 0.672, 0.789 for T1 CE, 0.774, 0.689, 0.700, 0.683 for FLAIR, as well as 0.861, 0.781, 0.778, 0.783 for the combination, respectively. The AUCs of radiologists 1, 2 and 3 were 0.700, 0.687, and 0.714, respectively. The efficacy of machine learning based on radiomics was superior to the radiologists’ assessment.

**Conclusions:**

Machine-learning based on radiomics of T1 CE and FLAIR offered superior efficacy to that of radiologists in differentiating ODG2 from ODG3.

## Background

Oligodendrogliomas (ODGs), predominantly occur in adults with a peak between 40 and 60 years of age, constitute 5–20% of all gliomas [1]. Patients with low-grade (ODG2) are slightly younger than those with high-grade, anaplastic tumors (ODG3) [2]. The co-deletion of the short arm of chromosome 1 (1p) and the long arm of chromosome 19 (19q) [3] occursin about 60–90% of ODGs, thus making it the molecular hallmark for ODGs [1].

Calcification [4, 5] and the cortical-subcortical location [5, 6], most commonly in the frontal lobe [4], are regarded as the characteristic features of ODGs. In contrast to other low-grade gliomas (LGG), minimal to moderate enhancement and moderately increased perfusion are commonly seen in ODGs, making the differentiation of OGD2 from OGD3 difficult. Besides, ODG3 often shares the imaging features with ODG2 on conventional MRI, leading to unreliable tumor grade prediction. Edema, haemorrhage, cystic degeneration and contrast enhancement are more commonly seen in ODG3, but may also be seen in ODG2 [4]. Thus, a new medical imaging diagnostic strategy for differentiation of ODG2 from ODG3 needs to be developed.

Advanced imaging techniques, including DWI, perfusion imaging, MR spectroscopy and PET, are employed to obtain more sensitive diagnostic markers, however with unsatisfying efficacy. Diffusion restriction is seldom observed in ODG2 [6]. Averaged ADC values are reported to be lower in high grade glioma (HGG) than in LGG, however, ADC values of ODG3 are overlapped with that of ODG2, making DWI unreliable maker to distinguish them [7]. Using the cut-off value of 1.75 for relative cerebral blood volume (rCBV) ratio, HGG can be differentiated from LGG with a sensitivity of 95% [8]. Unfortunately, these findings may not be suitable for differentiating ODGs, because markedly elevated rCBV can also be observed in ODG2, thus, a reliable distinction can’t be easily achieved [7, 9, 10]. This is due to the presence of the short capillary segments in ODGs [5] which may contribute to the relatively low specificity (70%) reported by Law et al. [8]. Therefore, focally elevated rCBV does not necessarily indicate ODG3. Besides, correlation of K^trans^ with tumor grade is even poorer than that of rCBV, and it is more commonly used to assess the treatment effects [11]. Taking together, the efficacies of advanced MRI techniques in differentiating ODG2 from ODG3 are limited.

Combining quantitative image features extracted from conventional T1-weighted contrast-enhanced (T1 CE) and fluid attenuated inversion recovery (FLAIR) images with machine learning algorithms, radiomics can provide comprehensive information that is difficult to perceive with visual inspection [12, 13] and is commonly used in tumor diagnosis, staging and prognosis of tumors [14–20]. However, most previous studies were mainly focused on advanced MR techniques, the varied post-processing models, varied interpretation and evaluation criteria restricted their clinical applications. Except for their limited diagnostic powers, these advanced MRI techniques are not commonly available in some rural areas. However, the T1 CE and FLAIR are widely-used in almost all hospitals as the image routine sequences for glioma diagnosis and staging. It is thus feasible to combine radiomics with T1 CE and FLAIR to establish a practical and economical imaging solution for differentiating ODG2 from ODG3.

In this study, we aimed to evaluate the diagnostic power of machine-learning based on T1 CE and FLAIR imaging radiomics in comparison with the radiologists’ performance in differentiating ODG2 from ODG3.

## Methods

### Patients

**Fig. 1:**
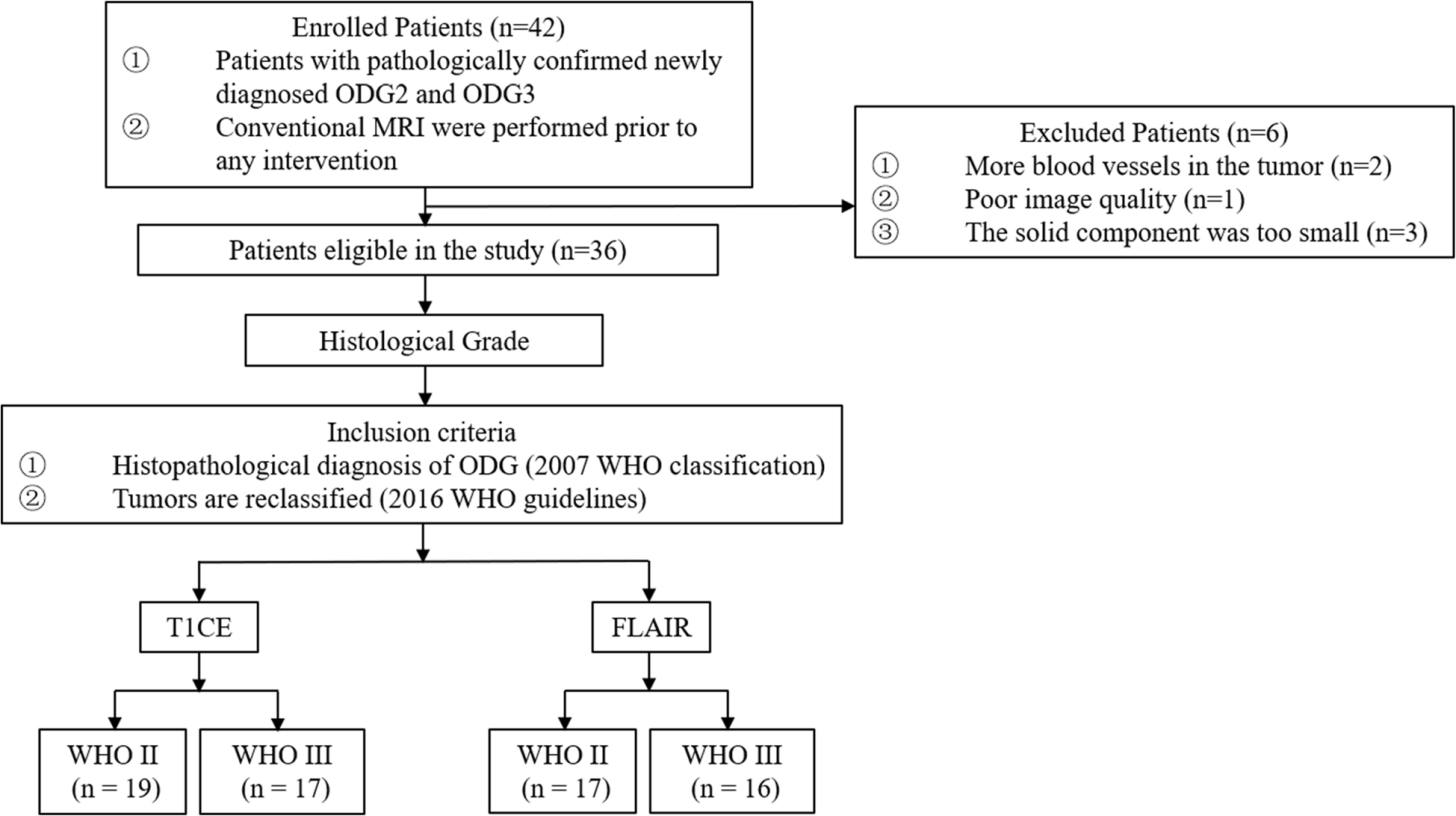
Flow diagram of the study design

This study was approved by our institutional review board and the requirement for informed consent was waived based on its retrospective nature. From January 2015 to July 2017, patients with confirmed ODGs were retrospectively and consecutively recruited. Tumors were classified according to 2007 WHO classification or 2016 WHO guidelines when enough information was available. The including criteria were, 1. patients underwent preoperative conventional MRI scan. 2. patients underwent gross total or subtotal tumor resection and a confirmative pathological diagnosis was made. Thirty-six patients with T1CE were included (19 men, 17 women; mean age = 45 years; age range = 9–65 years) and classified into two groups: ODG2 (*n* = 19; mean age = 46 years, age range = 10–65 years) and ODG3 (*n* = 17; mean age = 44 years, age range = 9–65 years). Thirty-three out of the above 36 patients with FLAIR were enrolled (18 men, 15 women; mean age = 45 years; age range = 9–65 years) and classified into two groups: ODG2 (n = 17; mean age = 45 years, age range = 10–65 years) and ODG3 (*n* = 16; mean age = 45 years, age range = 9–65 years). The patient selection is summarized in Fig. 1.

### MRI data acquisition

All patients underwent 3-T MR scanning (Discovery MR750, General Electric Medical System, Milwaukee, WI, USA) with an 8-channel head coil (General Electric Medical System). The initial routine scan sequences for each patient included T1-weighted imaging (T1WI) performed before and after contrast enhancement, an axial T2-weighted imaging (T2WI), and a transverse FLAIR to assist with diagnosis.

The parameters of the conventional MRI sequences were as the follows: T1WI with gradient echo (TR/TE, 1750 ms/24 ms; matrix size, 256 × 256; FOV, 24 × 24 cm; number of excitation, 1; slice thickness, 5 mm; gap, 1.5 mm), T2WI with turbo spin-echo (TR/TE, 4247 ms/93 ms; matrix size, 512 × 512; FOV, 24 × 24 cm; number of excitation, 1; slice thickness, 5 mm; gap, 1.5 mm) and sagittal T2WI (TR/TE, 10,639 ms/96 ms; matrix size, 384 × 384; FOV, 24 × 24 cm; number of excitation, 2; slice thickness, 5 mm; gap, 1.0 mm). We obtained axial FLAIR with the following parameters: TR/TE, 8000 ms/165 ms; matrix size, 256 × 256; FOV, 24 × 24 cm; number of excitations, 1; slice thickness, 5 mm; gap, 1.5 mm.

Finally, T1 CE were performed after intravenous bolus injection of gadodiamide (Omniscan; GE Healthcare, Co. Cork, Ireland), at a dose of 0.1 mmol/kg body weight. The parameters of T1 CE with volumetric interpolated breath-hold examination (VIBE) were as the follows: TR/TE, 8.2 ms/3.2 ms; T1, 450 ms; flip angle 12°; section thickness, 1.2 mm; FOV, 24 × 24 cm; matrix size, 256 × 256; number of excitations, 1; image number, 140.

### Tumor segmentation or delineation

Two neuroradiologists (S.S.Z with 8 years of experience and L.F.Y, with 12 years of experience in neuro-oncology imaging) independently reviewed all images. A third senior neuroradiologist (G.B.C, with 25 years of experience in euro-oncology imaging) re-examined the images and determined the final imaging diagnoses when inconsistency occurred. The preoperative conventional image features of tumor were retrieved based on the criteria outlined in Additional file 1**:** Table S1 (*online*).

The volumes of interest (VOIs) were semi-automatically segmented using ITK-SNAP (version3.6, http://www.itk-snap.org) by two neuroradiologists (S.S. Z and L.F.Y). The VOIs covering the enhanced lesion were drawn slice by slice on T1 CE and co-registered to and FLAIR images, avoiding the regions of macroscopic necrosis, cyst, edema and non-tumor macrovessels [21].

### Radiomics strategy

#### Feature extraction

Texture features include 162 first-order logic features, 216 Gy level co-occurrence matrix (GLCM) features, 144 Gy level run length matrix (GLRLM) features, 144 Gy level size zone matrix (GLSZM) features, 126 grey level difference matrix (GLDM) features, 45 neighborhood grey-tone difference matrix (NGTDM) features and 14 shape Features. A total of 1072 features were extracted from the T1 CE and FLAIR images using 3D-slicer software. We used the aforementioned features because these features were found to be relevant for distinguishing ODG2 from ODG3 in our previous studies by using MR imaging [16].

#### Feature selection

After being centered and scaled, the highly redundant and correlated features were subjected to a two-step feature selection procedure. First, highly correlated features were eliminated using Pearson correlation analysis, with the r threshold of 0.75. Then, a random forest (RF) classifier consisting of a number of decision trees was used to rank the feature importance. Every node in the decision trees is a condition on a single feature, designed to split the dataset into two so that similar response values end up in the same set. The measurement based on which optimal condition is chosen is called impurity. For classification, it is typically either Gini impurity or information gain/entropy. Thus, when training a tree, it can be computed how much each feature decreases the weighted impurity in a tree. To build the RF, the impurity decrease from each feature can be averaged and the features are ranked according to this measurement. In our study, Gini impurity decrease was used as the criterion to indicate the feature importance.

#### Radiomics model building

**Fig. 2:**
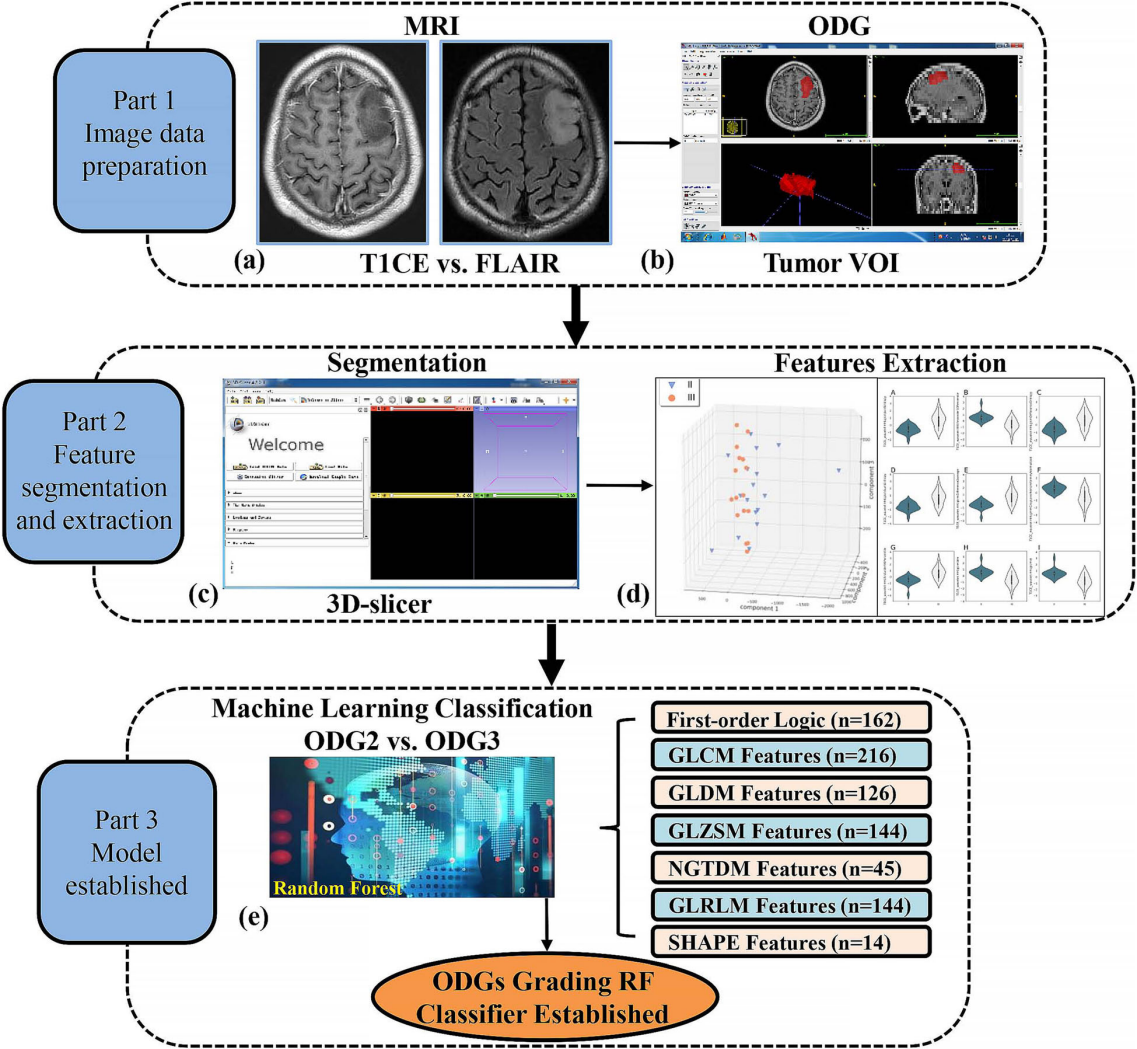
The main procedure of the radiomic strategy for preoperative ODGs grading. Based on T1 CE and FLAIR data (**a**) and tumor volume of interest (VOI) manually drawn on resampled T1 CE and FLAIR images (**b**), a group of parametric images are derived and the corresponding parametric maps of the whole tumor region are extracted (**c**). Utilizing radiomic features analysis; a big collection of tumor parameter attributes was acquired for the following machine learning process (**d**). Feature selection methods were implemented and compared using random forest (RF) classifier with additional discussion on model parameters to construct the optimal ODG grading model (**e**)

The 30 most important features were fed into a Conditional Inference RF classifier to build model [22]. Five-fold cross validation was employed for tuning hyper-parameter number of RF trees. Five-fold cross validation including pre-processing, feature selection and model construction were performed 3 times in order to avoid bias and overfitting as much as possible. The final results were the average from 3 performances. There was no feature selection in the combination of T1 CE and FLAIR throughout the model building. Accuracy, sensitivity and specificity were computed to evaluate the classifying performance. The receiver operating characteristic (ROC) curve was also built to provide the area under the ROC curve (AUC). The larger the AUC, the better the classification [23]. The whole procedure of feature extraction and machine learning was described in Fig. 2**.**

#### Radiologist’s assessment

To compare the efficacies of neuroradiologist and machine learning in differentiating ODG2 from ODG3, the images were also independently assess by three junior neuroradiologists (X.L.F, G. X and Y. H with 6, 7 and 7 years of neuroradiology experience, respectively). The neuroradiologists were blinded to the clinical information, but were aware that the tumors were either ODG2 or ODG3, without knowing the exact number of patients with each entity. The three readers assessed only conventional MR images (T1WI, T2WI, FLAIR and T1 CE), and recorded the final diagnosis using a 4-point scale (1 = definite ODG2; 2 = likely ODG2; 3 = likely ODG3; and 4 = definite ODG3) [24].

### Statistical analysis

Fisher exact test or the Chi-square test were used for the categorical variables and unpaired Student *t* test was used for continuous variable between ODG2 and ODG3 groups. The statistical analyses of clinical characteristics were performed by using SPSS 20.0 software (SPSS Inc., Chicago, IL, USA).

The statistical analyses of machine-learning were performed using R version 3. 4. 2 (R Foundation for Statistical Computing). A RF analysis was performed to train the machine-learning classifier. The goal of machine learning was to build the model to differentiate ODG2 from ODG3 based on radiomics features of T1 CE and FLAIR images. The following R packages were used: the random forest package was used for feature ranking; the caret and unbalanced packages were used for RF classification. Classifier performance was determined by using accuracy, sensitivity and specificity. The AUC values were also calculated for three readers and compared with that of the radiomics classifier. *P* value < 0.05 was considered as statistical significance.

## Results

### Patient characteristics

The main clinical characteristics and conventional MRI features of the 36 patients (ODG2 and ODG3) were summarized in Table 1. Tumor necrosis was more frequent in ODG3 than in ODG2 groups (*P* = 0.044), reflecting the hypoxia as a result of the rapid tumor growth. In addition, ODG3 were related to the nodular/ring-like enhancement patterns (*P* = 0.002). Besides, 10/19 (52.6%) of ODG2 and 10/17 (58.8%) of ODG3 situated in the frontal lobe, indicating no significant group difference. No significant difference of other clinical characteristics (gender, age) or imaging paradigms was observed between ODG2 and ODG3 patients.

**Table 1:** Clinical characteristics and MRI features of patients

Variable	ODG2	ODG3	Total	*P* value
No. of patients, n	19	17	36	NA
Location, n (%)				0.378
Frontal	10/19 (52.6)	10/17 (58.8)	20/36 (55.6)	
Temporal	3/19 (15.8)	5/17 (29.4)	8/36 (22.2)	
Parietal	3/19 (15.8)	1/17 (5.9)	4/36 (11.1)	
Insular	1/19 (5.3)	1/17 (5.9)	2/36 (5.6)	
Occipital	0/19 (0)	0/17 (0)	0/36 (0)	
Others	2/19 (10.5)	0/17 (0)	2/36 (5.6)	
Gender, n (%)				0.202
Male	8/19 (42.1)	11/17 (64.7)	19/36 (52.8)	
Female	11/19 (57.9)	6/17 (35.3)	17/36 (47.2)	
Age ^a^				0.788
Mean ± SD	45.6 ± 13.7	44.3 ± 15.1	45.0 ± 14.4	
Signal, n (%)				0.092
Homogeneous	6/19 (31.6)	1/17 (5.9)	7/36 (19.4)	
Heterogeneous	13/19 (68.4)	16/17 (94.1)	29/36 (80.6)	
Tumor cross midline, n (%)				1.000
No	16/19 (84.2)	14/17 (82.4)	30/36 (83.3)	
Yes	3/19 (15.8)	3/17 (17.6)	6/36 (16.7)	
Multiple foci, n (%)				0.736
No	12/19 (63.2)	9/17 (52.9)	21/36 (58.3)	
Yes	7/19 (36.8)	8/17 (47.1)	15/36 (41.7)	
Necrosis, n (%)				0.044*
No	13/19 (68.4)	5/17 (29.4)	18/36 (50.0)	
Yes	6/19 (31.6)	12/17 (70.6)	18/36 (50.0)	
Cyst, n (%)				0.255
No	16/19 (84.2)	11/17 (64.7)	27/36 (75.0)	
Yes	3/19 (15.8)	6/17 (35.3)	9/36 (25.0)	
Edema, n (%)				0.106
No	4/19 (21.1)	0/17 (0)	4/36 (11.1)	
Yes	15/19 (78.9)	17/17 (100.0)	32/36 (88.9)	
Border, n (%)				1.000
Sharp/smooth	2/19 (10.5)	1/17 (5.9)	3/36 (8.3)	
Indistinct/irregular	17/19 (89.5)	16/17 (94.1)	33/36 (91.7)	
Enhancement, n (%)				0.002*
No/blurry	15/19 (78.9)	4/17 (23.5)	19/36 (52.8)	
Nodular/ring-like	4/19 (21.1)	13/17 (76.5)	17/36 (47.2)	
Cognitive dysfunction, n (%)				0.274
No	7/19 (36.8)	3/17 (17.6)	10/36 (27.8)	
Yes	12/19 (63.2)	14/17 (82.4)	26/36 (72.2)	
Epileptic seizures, n (%)				1.000
No	10/19 (52.6)	9/17 (52.9)	19/36 (52.8)	
Yes	9/19 (47.4)	8/17 (47.1)	17/36 (47.2)	

### Quantitative MR histogram and texture features analysis

**Fig. 3:**
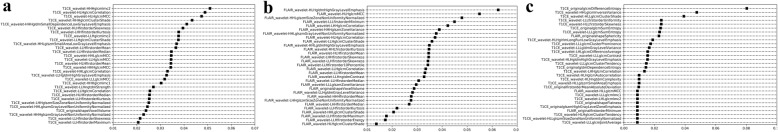
Feature importance plot shows mean decrease in Gini impurity. Features that most reduce Gini impurity are those that result in the least misclassification. Note: **a =** T1 CE; **b =** FLAIR; **c =** T1 CE + FLAIR

The relative importance of features computed by using the Gini index to differentiate ODG2 from ODG3 was depicted in Fig. 3. It can be seen that if all the high-throughput features were put into the RF classifiers, the classification performance could not be significantly improved because of the feature redundancy.

**Fig. 4:**
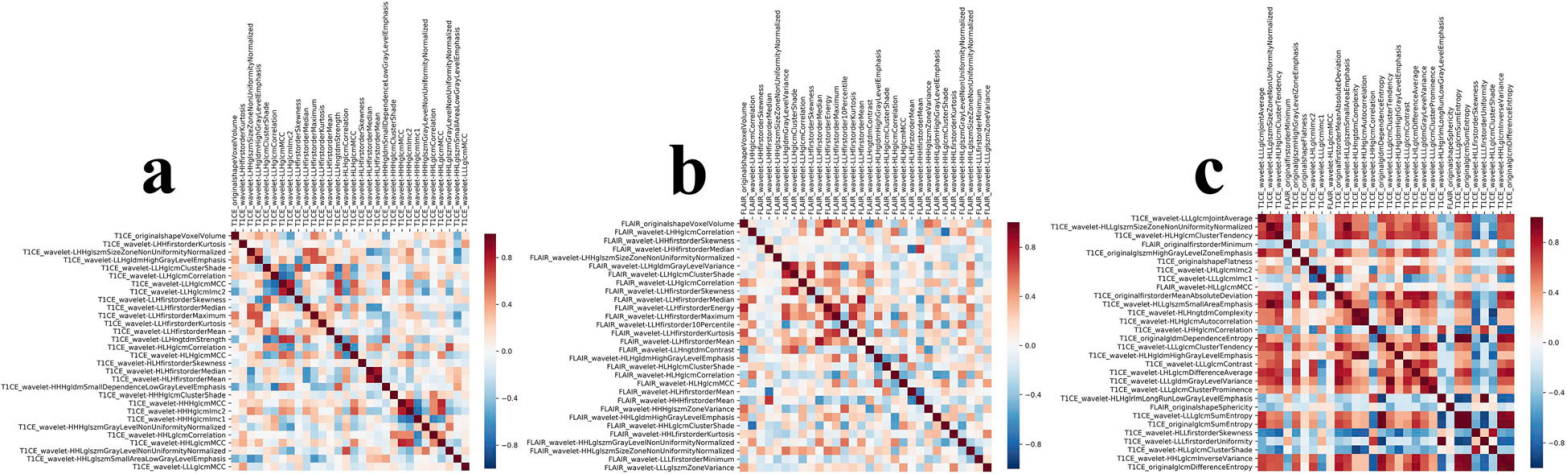
The radiomic heat map about the correlation analysis for feature selection: (**a**) T1 CE; (**b**) FLAIR; (**c**) T1 CE + FLAIR. **Note:** Red refers to positive correlations and blue refers to negative correlations. Different color depth indicates different values of correlation coefficients

The strong relationship between radiomic features to differentiate ODG2 from ODG3 was also indicated in the radiomic heat map (Fig. 4). The RF based feature selection strategy improved the performance of RF classifier. After RF feature selection, 30 optimal features were selected to differentiate ODG2 from ODG3, with comparable efficacy to that of using all features.

### Evaluation of principal components

**Fig. 5:**
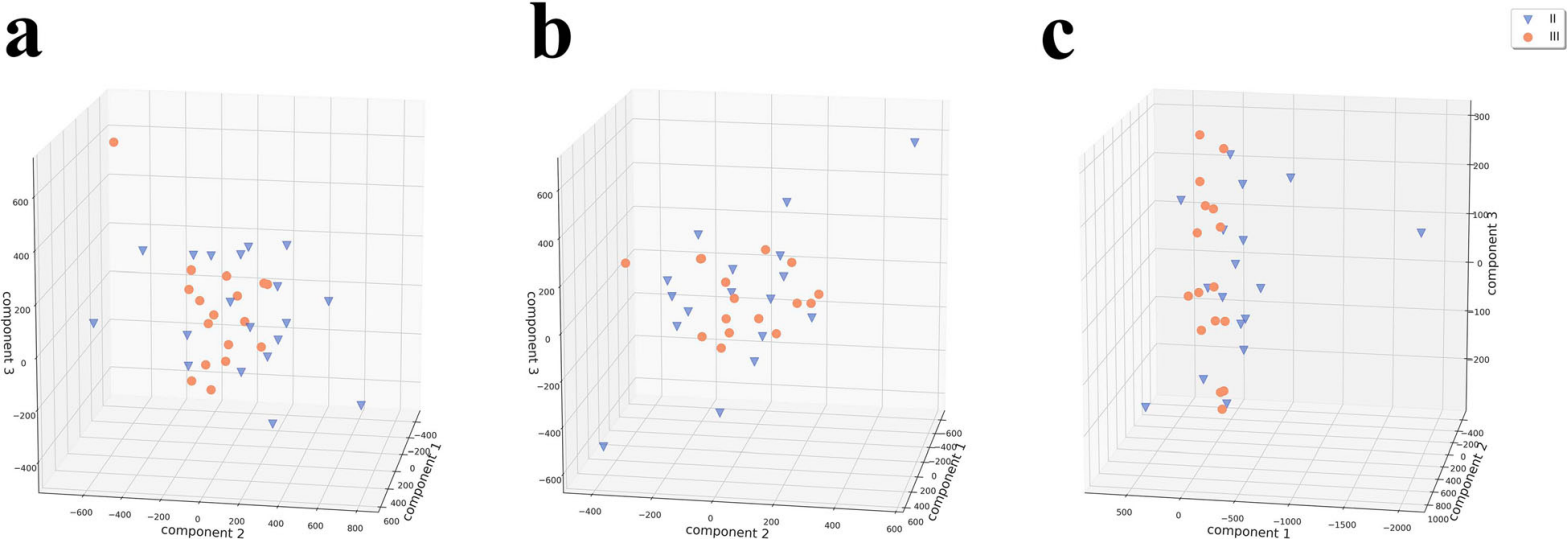
The calculated principal components for each tumor type were demonstrated based on the tumor tissue heterogeneity. II = ODG2, III = ODG3; component 1 = first principal component, component 2 = second principal component, component 3 = third principal component; **a =** T1 CE; **b =** FLAIR; **c =** T1 CE + FLAIR

When ODG2 and ODG3 were differentiated by using principal components, similar tumor tissue formed characteristic clusters. These clusters, although heterogeneous, defined a specific VOI (eg, Fig. 5) and were separable from other tumors (clusters). More important, the calculated principal components of the VOIs from ODG2 and ODG3 allowed clear separation of these two important regions.

### Diagnostic performance of radiomics and radiologists

**Fig. 6:**
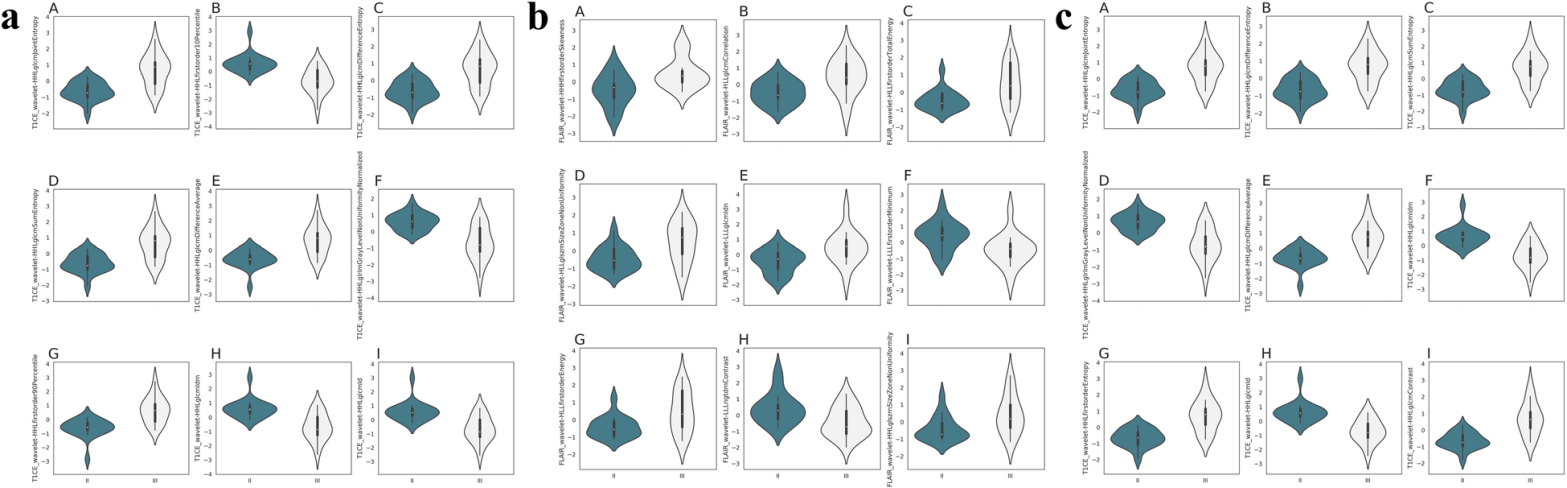
Violin plots show the values of first 9 radiomic features according to the grade of ODG. The small box in kernel density map represent the box plot. Points in small boxes = median values. Boundaries of small boxes = 25th and 75th percentiles. **a =** T1 CE; **b =** FLAIR; **c =** T1 CE + FLAIR. The violin represented kernel density map

**Fig. 7:**
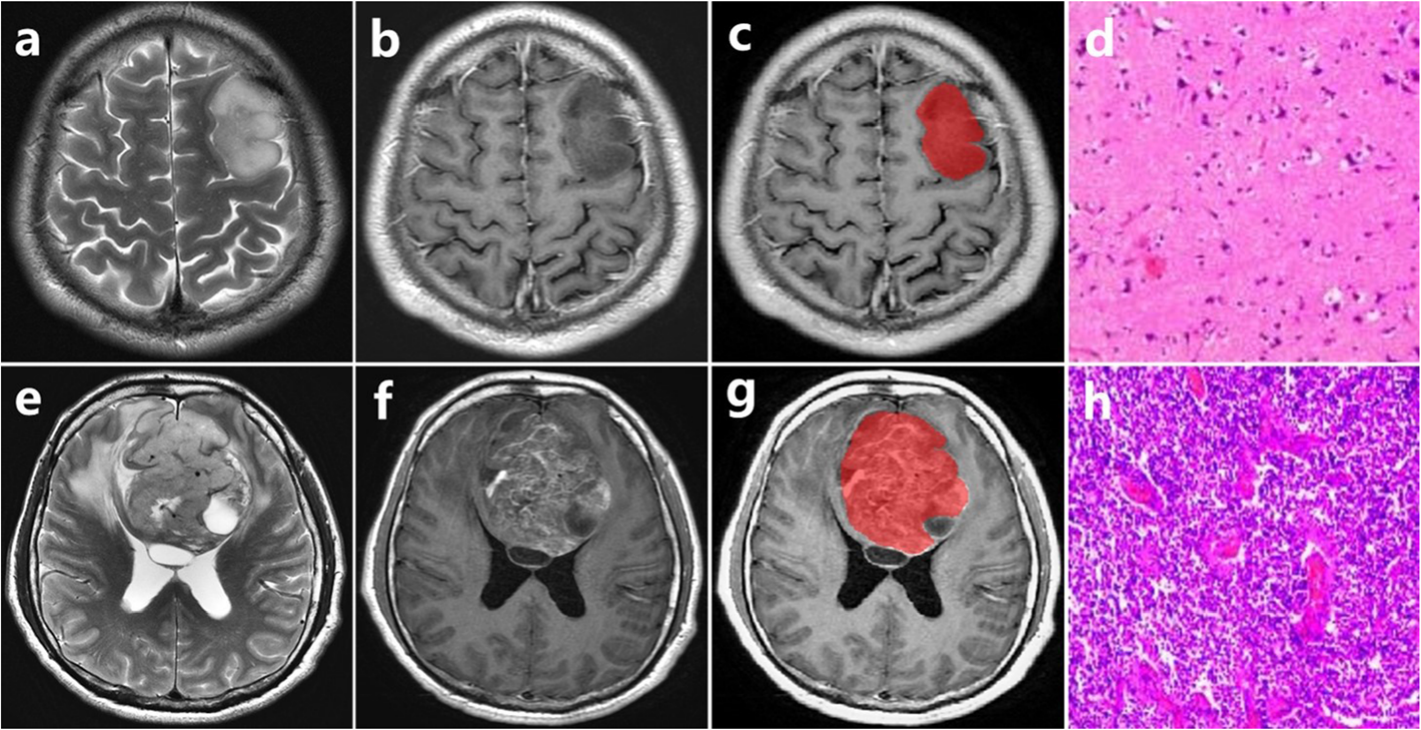
Upper row: ODG2 in the left frontal lobe from 33-year-old man; lower row: ODG3 in the bilateral frontal lobe from 46-year-old man. **a**, **e** T2-weighted image. **b**, **f** T1-weighted contrast-enhanced image. **c**, **g** The volume of interest of manually drawn. **d**, **h** Pathology slice images show cell density and vascular proliferation

The performance of radiomics and 3 radiologists in differentiating ODG2 from ODG3 was also compared. Table 2 and Fig. 6 summarized the diagnostic performance of the radiomic features derived by using MR images from T1 CE, FLAIR and their combination to distinguish ODG2 from ODG3. Radiomic features from their combination showed significantly better diagnostic performance than that of FLAIR or T1 CE. Violin plots graphed for the first 9 radiomic features derived from T1 CE, FLAIR and their combination were presented in Fig. 6. The AUC, sensitivity, specificity and accuracy of radiomics were 0.798 (95%CI 0.699–0.896), 0.672, 0.789, 0.735 for T1 CE, 0.774 (95%CI 0.671–0.877), 0.700, 0.683, 0.689 for FLAIR, and 0.861 (95%CI 0.783–0.940), 0.778, 0.783, 0.781 for their combination, respectively. The AUCs of the three radiologists were 0.700 (95%CI 0.519–0.880), 0.687 (95%CI 0.507–0.867) and 0.714 (95%CI 0.545–0.883) for readers 1, 2 and 3, respectively. The radiomics classifier performed superior to the 3 junior radiologists. The representative cases of ODG2 and ODG3 were presented in Fig. 7. The clinical application of radiomics-based machine learning could be justified based on our findings.

**Table 2:** Diagnostic performance of comparison of radiomics and human assessment

	Sensitivity	Specificity	AUC	ACC
Radiomics (T1 CE)	0.672	0.789	0.798 (95% CI: 0.699, 0.896)	0.735
Radiomics (FLAIR)	0.700	0.683	0.774 (95% CI: 0.671, 0.877)	0.689
Radiomics (T1 CE + FLAIR)	0.778	0.783	0.861 (95% CI: 0.783, 0.940)	0.781
Reader1	0.824	0.632	0.700 (95% CI: 0.519, 0.880)	0.722
Reader2	0.706	0.684	0.687 (95% CI: 0.507, 0.867)	0.694
Reader3	0.647	0.632	0.714 (95% CI 0.545–0.883)	0.667

## Discussion

Radiomics is an emerging field that treats images as data rather than pictures and analyzes a large number of features extracted from 1 image in relation to clinical variables of interest. A few studies on radiomics analyses of glioma have been published over the last years and advocated for machine learning models in predicting tumor histology and grade [25]. Radiomics has been suggested as a robust strategy to noninvasively classify lesions [14, 26]. This work suggested that radiomics from T1 CE and FLAIR can be useful for differentiating ODG2 from ODG3, with the superior efficacy to that of radiologists, thus, its clinical application could be justified based on the current study.

From the angle of experiment design, there are three aspects worthy noting in this study. First, the ‘real world’ data were used to test our scientific hypothesis. Second, all images analyzed in the current study were taken exclusively from routine clinical diagnostic scans. Third, based on the social-economic consideration, the levels of accuracy were based on the radiomics of commonly available T1 CE and FLAIR images, without an acquisition of spectroscopy, CBV or perfusion information, all of which would prolong the scanning time and increase economic burden to patients. Upon our expectation, the radiomic strategy performed superior to that of radiologists.

The reasons for the improved diagnostic performance of radiomics are as the following. First, radiomic methods, given their ability to discern patterns and combine information in a way that humans cannot, showed substantial promise for the future of radiology and precision medicine [27]. However, radiologists distinguished ODG2 from ODG3 by visual diagnosis using rough information from T1 CE and FLAIR. Second, it has been reported that the performance of an SVM classifier can be significantly reduced by the inclusion of redundant features and this effect is more obvious for a small training set [28]. In this study, it was found that the combination of conventional T1 CE and FLAIR features provided lower classification error than features of individual sequence, which may thus emphasize the importance of using a multiparametric approach. In addition, highly correlated features were eliminated using Pearson correlation analysis, which was also further ranked by using the random forest classifier consisting of a number of decision trees. This indicated that redundant features removed can have a contribution to the classification of ODG2 and ODG3.

Radiomic strategy not only performed superior to radiologists, but also could be used as an auxiliary means to overcome some problems attained to radiologists. First of all, the frequency of interruptions during a reporting session is associated with up to 13% increase in time for reporting and an increased potential for errors [29]. Then, fatigue adversely impacts the visual system including: worse accommodation, decreased saccadic velocity and reduced gaze volume and coverage [30]. At last, a number of cognitive biases may adversely affect the accuracy of a radiologists report of a glioma [31]. In order to reduce reporting time and cognitive biases, both of which may lead to reporting and diagnostic errors, radiomics offers a significant advantage [32], particularly in the context of general radiologists who may lack expertise in neuro-oncology. Nevertheless, the current radiomic strategy involves too much pre- and post-process before the suitable machine learning model is established, more studies focusing on the efficacy-cost balance of such a machine learning system should be further conducted before its clinical application.

Furthermore, a few limitations of this study should be noticed. In the first place, sample number of the patients is relatively small. Although current results of 5-fold cross validation showed that the evaluation of diagnostic efficacy were robust despite the relatively small sample size, which did not cause the classifier to be skewed towards a particular class. It is desirable to verify the classifier on a larger data size in the future. Besides, this radiomic method incorporated vessel removal in its methodology, this method may fail for certain cases that were non-tumor vessels intertwined with tumor vessels. Signal intensity curves of prominent vessels can be used as a differentiating feature for such cases.. The last, a continuous effort on enlarging the dataset so as to test its external validation is required.

## Conclusions

In conclusion, this study demonstrates our findings that use of a machine learning algorithm, derived from ‘real word’ T1 CE and FLAIR images, which can differentiate ODG2 from ODG3 in newly diagnosed gliomas with a superior efficacy to that of radiologists. The RF selected features can reduce the labor in applying this strategy, and the strategy can be applied clinic based on our findings.

## Supplementary information

**Additional file 1 : Table S1.** Image definition

## Data Availability

The datasets used and/or analysed during the current study are available from the corresponding author on reasonable request.

## Abbreviations

BBB: Brain Blood Barrier
FLAIR: Fluid-Attenuated Inversion ecovery
HGG: High Grade Glioma
LGG: Low Grade Glioma
ODG: Oligodendroglioma
rCBV: Relative Cerebral Blood Volume
RF: Random Forest
T1 CE: T1-weighted contrast-enhanced image
T1WI: T1-weighted imaging
T2WI: T2-weighted imaging
