# Supplementary material for: Better efficacy in differentiating WHO grade II from III oligodendrogliomas with machine-learning than radiologist’s reading from conventional T1 contrast-enhanced and fluid attenuated inversion recovery images
Source: BMC Neurol. 2020 Feb 7;20:48. doi: 10.1186/s12883-020-1613-y (PMC7007642; doi:10.1186/s12883-020-1613-y)
Supplement: Supplementary file 1 — Additional file 1 : Table S1. Image definition [file 12883_2020_1613_MOESM1_ESM.docx]

**Supplementary Table 1** Image definition

| Variable | Classification criteria |
| --- | --- |
| Location |  |
| Frontal | Major tumor mass in the frontal lobe, one or more regions of tumor, either enhancing or non-enhancing |
| Temporal | Major tumor mass in the temporal lobe, one or more regions of tumor, either enhancing or non-enhancing |
| Parietal | Major tumor mass in the parietal lobe, one or more regions of tumor, either enhancing or non-enhancing |
| Insular | Major tumor mass in the insular lobe, one or more regions of tumor, either enhancing or non-enhancing |
| Occipital | Major tumor mass in the occipital lobe, one or more regions of tumor, either enhancing or non-enhancing |
| Others | Major tumor mass in other parts but not the lobes of the brain, one or more regions of tumor, either enhancing or non-enhancing |
| Signal |  |
| Homogeneous | Only one signal of tumor |
| Heterogeneous | More than or equal to 2 signals of tumor, including cyst, [hemorrhage](http://dict.youdao.com/w/hemorrhage/#keyfrom=E2Ctranslation) or necrosis, etc |
| Tumor cross midline |  |
| No | Tumor is limited to the unilateral cerebral hemisphere |
| Yes | Tumor crosses the brain midline and extends into contralateral cerebral hemisphere |
| Multiple foci |  |
| No | Only one region of tumor, either enhancing or non-enhancing |
| Yes | More than or equal to 2 regions of tumor, either enhancing or non-enhancing, which is not contiguous with the major tumor mass |
| Necrosis |  |
| No | No necrosis in the tumor |
| Yes | Necrosis in the tumor |
| Cyst |  |
| No | No cyst within the tumor |
| Yes | Cyst within the tumor |
| Edema |  |
| No | No convincing edema |
| Yes | Definite Edema extending from tumor margin |
| Border |  |
| Sharp/smooth | Sharp/smooth from tumor boundaries |
| Indistinct/irregular | Indistinct/irregular from tumor boundaries |
| Enhancement |  |
| No/blurry | No/blurry enhancement from tumor |
| Nodular/ring-like | Nodular/ring-like enhancement from tumor |
